# Pathogenic Role of MicroRNA Dysregulation in Podocytopathies

**DOI:** 10.3389/fphys.2022.948094

**Published:** 2022-06-29

**Authors:** Feng Liu, Jiefang Chen, Changqing Luo, Xianfang Meng

**Affiliations:** ^1^ Department of Nephrology, Union Hospital, Tongji Medical College, Huazhong University of Science and Technology, Wuhan, China; ^2^ Department of Neurology, Union Hospital, Tongji Medical College, Huazhong University of Science and Technology, Wuhan, China; ^3^ Department of Neurobiology, Institute of Brain Research, School of Basic Medical Sciences, Tongji Medical College, Huazhong University of Science and Technology, Wuhan, China

**Keywords:** microRNA, podocytopathy, therapeutic target, focal segmental glomerulosclerosis, minimal change disease, membranous nephropathy, diabetic kidney disease, IgA nephropathy

## Abstract

MicroRNAs (miRNAs) participate in the regulation of various important biological processes by regulating the expression of various genes at the post-transcriptional level. Podocytopathies are a series of renal diseases in which direct or indirect damage of podocytes results in proteinuria or nephrotic syndrome. Despite decades of research, the exact pathogenesis of podocytopathies remains incompletely understood and effective therapies are still lacking. An increasing body of evidence has revealed a critical role of miRNAs dysregulation in the onset and progression of podocytopathies. Moreover, several lines of research aimed at improving common podocytopathies diagnostic tools and avoiding invasive kidney biopsies have also identified circulating and urine miRNAs as possible diagnostic and prognostic biomarkers for podocytopathies. The present review mainly aims to provide an updated overview of the recent achievements in research on the potential applicability of miRNAs involved in renal disorders related to podocyte dysfunction by laying particular emphasis on focal segmental glomerulosclerosis (FSGS), minimal change disease (MCD), membranous nephropathy (MN), diabetic kidney disease (DKD) and IgA nephropathy (IgAN). Further investigation into these dysregulated miRNAs will not only generate novel insights into the mechanisms of podocytopathies, but also might yield novel strategies for the diagnosis and therapy of this disease.

## Introduction

Podocytes are highly specialized, terminally differentiated glomerular visceral epithelial cells that are indispensable for the maintenance of an of intact glomerular filtration barrier in the kidney (Mathieson, 2011). Due to the restricted proliferation capacity, podocytes are especially vulnerable to a series of injurious stimuli, such as hyperglycemia, transforming growth factor β (TGF-β), angiotensin II, as well as adriamycin and puromycin, which could ultimately result in podocytes loss (Kang et al., 2010; Zhou and Liu, 2015). Glomerular podocytes are the primary target in majority of glomerular diseases (Arif et al., 2019), mainly including diabetic kidney diseases (DKD), focal segmental glomerulosclerosis (FSGS), minimal change disease (MCD), membranous nephropathy (MN), and IgA nephropathy (IgAN), leading to consequent albuminuria or proteinuria and subsequent renal function decline (Webster et al., 2017; Torban et al., 2019). Thus, preventing or reversing podocyte injury is an important strategy to treat podocyte-associated diseases. In the past decades we have witnessed dramatic advances in the understanding of podocyte biology as well as molecular mechanisms involved in podocyte injury (Mathieson, 2011; Lazzeri and Romagnani, 2015; Zhou and Liu, 2015; Kopp et al., 2020). Nevertheless, developing podocyte-specific targeted therapeutic strategies is still a great challenge. Currently, many pharmaceutical agents such as calcineurin inhibitors (CNIs), glucocorticosteroids (GCS), and mTOR inhibitors (mTORIs) have been reported to have protective effects against the podocyte injury. However, the side effects due to the non-specific nature of those agents pose a serious concern in the clinical practice (Mathieson, 2011; Kopp et al., 2020). Thus, identifying the key molecules that are implicated in different types of podocytopathies might offer important clues for the development of new therapeutic strategies for treating people with proteinuric kidney disease.

MicroRNAs (miRNAs) are a class of non-coding RNAs (ncRNAs) with an average length of 22 nucleotides, which have been first discovered by Lee et al., in 1993 (Lee et al., 1993; Yates et al., 2013). Although miRNAs do not have the ability to encode proteins, they are able to control the expression of their target genes at the post-transcriptional level (Borchert et al., 2006; Krol et al., 2010). Functionally, miRNAs mainly bind to the miRNA response elements (MREs) in the 3′-untranslated region (3′-UTR) of their target mRNAs, leading to mRNA degradation and/or mRNA translational inhibition (Borchert et al., 2006; Filipowicz et al., 2008; Krol et al., 2010; Yates et al., 2013). It has been predicted that over 60% of the human protein-coding genes are regulated by miRNAs (Friedman et al., 2009). Accordingly, as miRNAs are involved in the modulating the expression of entire gene networks, dysregulation of certain miRNAs can cause or contribute to a wide variety of human diseases (Gebeshuber et al., 2013; Badal et al., 2016; Treiber et al., 2019; Agbu and Carthew, 2021; Schober et al., 2021; Liu et al., 2022; Zhou et al., 2022). In addition to their critical roles in modulating gene expression, miRNAs have also been clinically used as promising non-invasive diagnostic, prognostic, and predictive biomarkers for several human diseases, such as malignant pleural mesothelioma, acral melanoma, clear cell renal carcinoma, HBV-related hepatocellular carcinoma and acute myocarditis (van Zandwijk et al., 2017; Treiber et al., 2019; Hong et al., 2020; Blanco-Domínguez et al., 2021). Previous studies have also indicated that miRNAs play pivotal roles in the development and progression of various glomerular diseases associated with podocyte dysfunction (Kato et al., 2009; Gebeshuber et al., 2013; Lazzeri and Romagnani, 2015; Trionfini and Benigni, 2017), indicating that they might also represent potential biomarkers and therapeutic targets for the diagnosis and treatment of podocytopathies. Moreover, conditional knockout of Dicer or Drosha, the key enzymatic regulators of miRNAs biogenesis and maturation, in a podocyte-specific manner results in progressive proteinuria and glomerulosclerosis, suggesting that miRNAs are also important genomic regulators of podocyte homeostasis (Shi et al., 2008; Zhdanova et al., 2011). Yet, the exact molecular mechanisms by which miRNAs modulate podocyte injury in podocytopathies still remains to be fully elucidated.

Although a large number of studies have demonstrated the critical roles of miRNAs in the occurrence and development of various podocytopathies, to the best of our knowledge, no previous systematic review has been performed to systematically generalize the involvement of miRNA dysregulation in podocytopathies. This review will mainly focus on the current understanding of pathogenic roles of miRNA dysregulation that links to podocyte injury in various podocytopathies and shed light on their use as potential biomarkers and treatment targets for this disease, which would benefit from further research.

## Podocytopathies

### Epidemiology and Etiology of Podocytopathies

**FIGURE 1:**
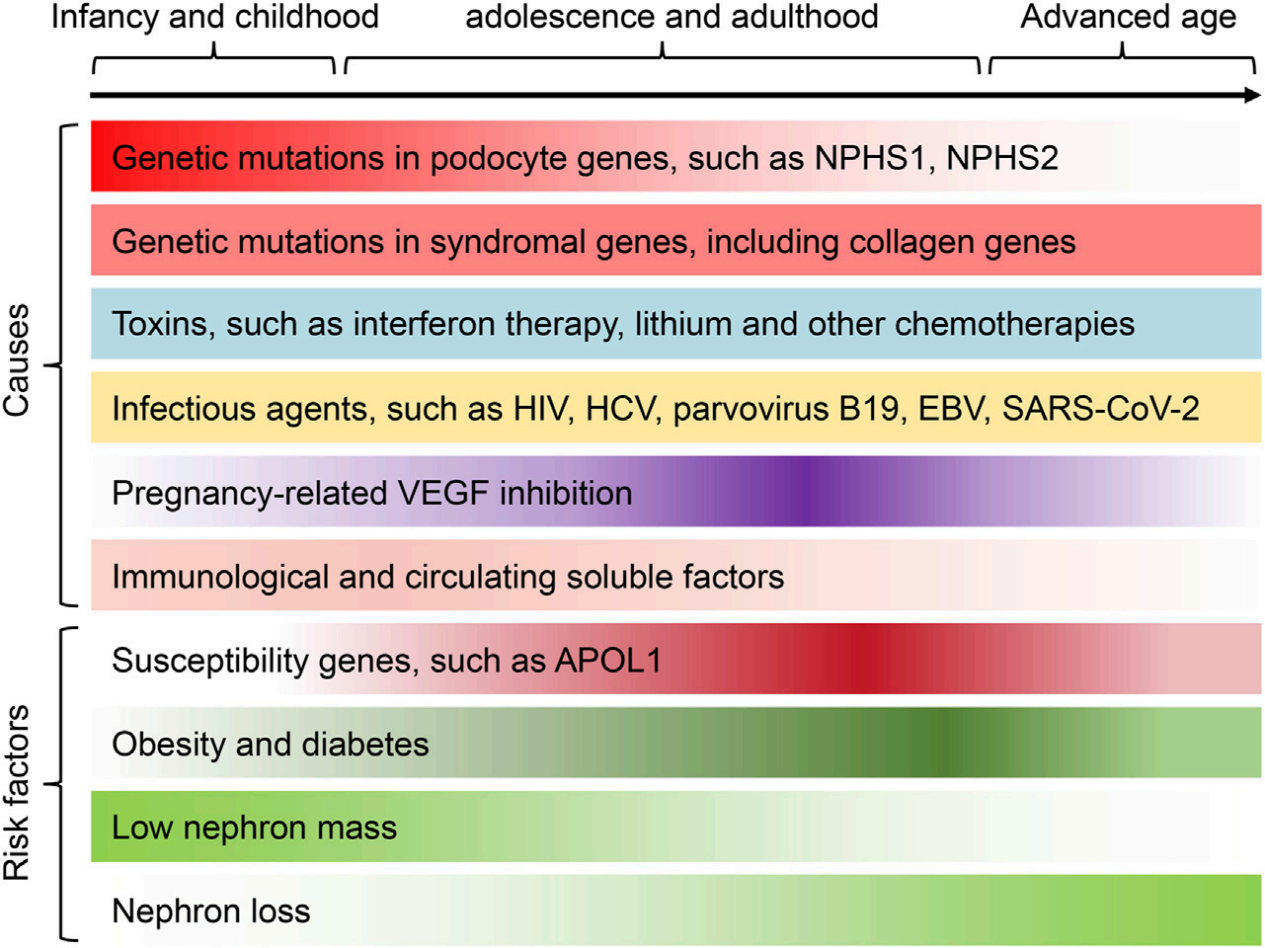
The underlying causes and risk factors of podocytopathies across the lifespan. A number of causes and risk factors have been revealed, mainly including genetic factors, immunological and/or soluble factors, VEGF inhibition, adaptive podocyte stress, infectious agents and various toxins, that predispose individuals to development of podocytopathies. Different risk factors and/or causes of podocytopathies can present at certain phases of life or be preferentially associated with gender and race. For instance, genetic causes of podocytopathies are more frequent in children and young adults. Podocytopathies associated with VEGF inhibition are more common in pregnant women. Among the major risk factors leading to the development of podocytopathies, nephron loss, severe obesity and diabetes are more frequently observed in adult middle-age patients, whereas low nephron mass is more frequent in adolescence or early adulthood. Besides, the susceptibility gene APOL1 is more prevalent in patients of Black adult. Finally, podocytopathies induced by various toxins and infectious agents can occur at all ages. The color gradient in each cause and risk factor represents the incidence of podocytopathies at different ages. APOL1, apolipoprotein L1; HCV, hepatitis C virus; EBV, Epstein–Barr virus; SARS-CoV-2, the virus that causes COVID-19.

Currently, despite the high incidence of podocytopathies, reliable epidemiological data on podocytopathies are still lacking (Kopp et al., 2020). The pathological diagnosis of podocytopathies is predominantly based on kidney biopsy, but many patients are not suitable for biopsy or lack of sufficient resources to perform biopsy, leading to an underestimation of the incidence of podocytopathies. In spite of the limitation, the prevalence of podocytopathies seems to be increasing globally, which is a leading cause of the increased prevalence of end-stage kidney disease (ESKD) (Rosenberg and Kopp, 2017). Currently, a number of key causes and risk factors have been revealed (Figure 1), mainly including genetic factors (Qiu et al., 2018), obesity (D'Agati et al., 2016), diabetes (Tonneijck et al., 2017), low nephron mass and nephron loss (Luyckx et al., 2017), immunological and/or soluble factors (Iijima et al., 2014; Kim et al., 2017; Colucci et al., 2019), vascular endothelial growth factor (VEGF) inhibition (Craici et al., 2014; Ollero and Sahali, 2015), infectious agents (Cohen et al., 2017; Nasr and Kopp, 2020) and various toxins (Rosenberg and Kopp, 2017; Puelles et al., 2019), that predispose individuals to development of podocytopathies. Alternatively, podocytopathies can be caused by a combination of diverse genetic and/or environmental risk factors that lead to podocyte damage, acting together to achieve a threshold effect for the development of proteinuria.

### Mechanisms and Pathophysiology of Podocytopathies

As terminally differentiated epithelial cells, the primary and secondary processes of podocytes cover the outer side of the glomerular basement membrane (GBM), constituting the glomerular filtration barrier (Wiggins, 2007; Mathieson, 2011). Foot process effacement (FPE) of podocytes is the earliest morphological patterns of podocyte injury, which is closely related to different degrees of proteinuria in the progress of various podocytopathies (Wiggins, 2007; Kopp et al., 2020). Although the effacement of podocytes foot process can be invertible, podocyte detachment from the GBM or death indicates inevitable podocyte loss, which can be mainly attributed to different unfavorable factors such as genetics, metabolism, toxicity or inflammation (Mundel and Shankland, 2002; Mundel and Reiser, 2010). Following damaged podocytes loss, the remaining podocytes adapt to expand to cover the newly denuded GBM, resulting in hypertrophy of remaining podocytes (Shankland, 2006). In addition, parietal epithelial cells (PECs), located in the inner portion of the Bowman capsule, are resident podocyte progenitors that can provide new podocytes following injury and loss (Ronconi et al., 2009; Eng et al., 2015). Despite these mechanisms play a beneficial role in the restoration of podocyte function and decrease proteinuria, they may be maladaptive or inefficient. Indeed, the capacity of hypertrophic podocytes is limited, which may be incapable to maintain the normal structure of podocyte foot processes, leading to enhanced local shear stresses that may subsequently trigger the process of podocyte detachment (Shankland, 2006; Fukuda et al., 2012). Furthermore, the differentiation of PECs into podocytes can be impaired by unfavorable factors (such as mechanical stress and proteinuria), ultimately resulting in inefficient podocyte regeneration or focal scar formation (Peired et al., 2013; Kopp et al., 2020). Nevertheless, despite certain progress has been achieved in exploring the molecular mechanisms and pathophysiology of podocytopathies, we are still far from fully understanding the pathogenesis of podocytopathies.

### Diagnosis and Treatment of Podocytopathies

Currently, the diagnosis of podocytopathies is predominantly based on clinical manifestations combined with pathological findings. Renal biopsy is routinely conducted on all adults with nephrotic-range proteinuria to guide treatment and provide prognosis (Mathieson, 2011; Kopp et al., 2020). However, the decision whether and when to perform a kidney biopsy in children with persistent non-nephrotic proteinuria is still controversial (Hama et al., 2012; Leung et al., 2017). A detailed differential diagnosis of podocytopathies may demand a combination of personal and family history, clinical manifestations, histopathological testing, serological inspection, imaging examination and/or genetic analyses (Wiggins, 2007; Kopp et al., 2020). Podocytopathies can be regarded as a consequence of podocyte dysfunction induced by various genetic and/or environmental elements. The clinical syndromes associated with podocyte dysfunction mainly include the following several types: DKD, FSGS, MCD, MN, Alport’s syndrome, hypertensive, nephropathy (HTN), immune and inflammatory glomerulonephropathies (Imm/Inf GN) (Wiggins, 2007; Nagata, 2016).

Therapeutic approaches in the management of patients with podocytopathies presenting with massive proteinuria or nephrotic syndrome can vary depending on the population served and available resources. Children and adult patients with consistent non-nephrotic proteinuria are primarily managed with dietary salt restriction and renin-angiotensin system inhibitors (RASI) (Mathieson, 2011; Ayalon and Beck, 2015). Besides, low-dose thiazide diuretic will have an additive effect on the anti-proteinuric effect. Children and adolescent patients with new onset nephrotic syndrome in the absence of histological verification are usually treat with oral steroid therapy for two to three months (Zhao and Liu, 2020). Nevertheless, due to the heterogeneous clinical behavior and response to treatment with steroid, the dose and treatment duration for the steroid should be adjusted appropriately according to clinical response. In adult patients, kidney biopsy, laboratory inspections as well as renal imaging examinations preclude other glomerular disorders, thereby guiding the management of podocytopathies based on the underlying etiologies. Glucocorticoids are the first choice for the treatment of some cases with no specific cause can be ascertained (Kopp et al., 2020; Medjeral-Thomas et al., 2020). Patients with sub-nephrotic proteinuria are usually managed with maximal doses of RASI and followed longitudinally according to renal function and residual proteinuria. However, increasing evident suggests that, beyond the basic initial steroid therapy, a substantial proportion of patients with podocytopathies are in an urgent need of a personalized treatment plan to prevent unnecessary drug-related toxicity. Thus, more in-depth research is needed to get a systemic understanding of the underlying mechanisms of podocytopathies, so as to provide novel therapeutic strategies to combat this type of disease.

### Role of MiRNAs in Podocytopathies

Increasing evidence indicates that miRNAs are critical regulators of gene expression in mammals, and the dysregulation of miRNAs is a common feature of various human diseases, including podocytopathies (Kato et al., 2009; Trionfini and Benigni, 2017; Fan et al., 2020). In the following sections, we will mainly focus on and summarize the current knowledge on the implications of miRNAs in the occurrence and development of podocytopathies as well as their potential value of clinical application in the diagnosis, treatment and prognosis of this disease.

### MiRNAs in Focal Segmental Glomerulosclerosis

Focal segmental glomerulosclerosis (FSGS) is a group of clinicopathological syndromes sharing a common glomerular lesion and characterized by marked proteinuria and podocyte injury, which is the result of diverse insults directed to or inherent within the podocyte (D'Agati et al., 2011; Wei et al., 2011; Fogo, 2015). A broad range of factors such as genetic, virus infections, drugs, and circulating factors can contribute to the pathogenesis of FSGS, which can be broadly classified into primary (idiopathic) and secondary forms, with approximately 80% of cases being primary (D'Agati et al., 2011). Current therapeutic strategies for FSGS mainly focus on reduction of urinary protein excretion and preserving renal function (D'Agati et al., 2011; Rosenberg and Kopp, 2017). Approximately 40% of patients with primary FSGS who receive kidney transplantation eventually develop recurrent disease in the allograft (D'Agati et al., 2011). Thus, further elucidation of circulating-factor caused podocyte damage as well as understanding the mechanisms of podocyte stabilization may help to shed new light on the specific pathogenetic mechanisms involved in primary FSGS.

Recent years have witnessed a flourishing interest in exploring the underlying role of miRNAs in FSGS (Gebeshuber et al., 2013; Trionfini and Benigni, 2017). For example, Gebeshuber and colleagues performed the first study to investigate the role of specific miRNAs in FSGS (Gebeshuber et al., 2013). In this study the researchers originally identified miR-193a can be highly inducible by doxycycline in miR-193a transgenic mice under the control of a cytomegalovirus (CMV)-tet promotor, whereas the upregulation of miR-193a in transgenic mice induced by doxycycline resulted in rapidly progressing FSGS and death from kidney failure within 12 weeks in heterozygous mice and 6 weeks in homozygous mice, respectively. Mechanistical studies revealed that miR-193a may suppress the expression of the Wilms’ tumor protein (WT1), a transcription factor that is indispensable for maintaining podocyte differentiation and homeostasis. They concluded that the enhanced expression of miR-193a in FSGS unveiled a novel pathogenic mechanism for FSGS and may provide a novel approach to treat FSGS. Consistent with those findings, a recent study demonstrated that higher miR-193a level in urinary exosomes was related to higher probability of primary FSGS and indicated poor prognosis in children with nephrotic syndrome (Wang et al., 2021). In another study, the association between urinary miRNAs levels and disease activity and treatment response in patients with FSGS was also reported (Zhang et al., 2014). In order to identify urinary miRNA-based biomarkers for FSGS disease activity as well as predict patients responds to steroid therapy, Zhang and his colleagues performed miRNA profiling among patients with active FSGS (FSGS-A), FSGS in remission (FSGS-CR), and age- and sex-matched normal controls. A total of 54 candidate miRNAs were identified, among which the levels of miR-490, miR-196a, and miR-30a-5p in urine samples were confirmed to be significantly upregulated in FSGS-A patients compared with normal controls and FSGS-CR patients and could be used as sensitive indicators of disease activity. In addition, the same research group also found that plasma miR-186 level was significantly upregulated in FSGS patients with nephrotic proteinuria and declined with disease remission, which was positively correlated with urinary protein levels in patients with FSGS, implying the possibility of miR-186 as a specific biomarker for FSGS (Zhang et al., 2015). Whereas, another study showed that the miR-30 family were highly expressed in podocytes and downregulated in patients with FSGS, which protected podocytes against injury induced by deleterious factors (TGF-β, PAN, or LPS) through targeting Notch1 and p53 (Wu et al., 2014). And exogenous overexpression of miR-30a attenuated proteinuria and podocyte injury in PAN-treated rats (Wu et al., 2014). Wu et al. also demonstrated that miR-30s were consistently downregulated in podocytes of FSGS patients and PAN-treated rats, and identified that downregulation of miR-30s results in calcium/calcineurin signaling activation, thus leading to podocyte injury in FSGS (Wu et al., 2015). In addition, other studies have also reported the involvement of some other dysregulated miRNAs in FSGS, such as miR-135a (Yang et al., 2017), miR-155 (Ramezani et al., 2015), miR-206 (Guo et al., 2016), miR-150 (Qi et al., 2020), miR-106a (Xiao et al., 2018) and miR-146-5p (Williams et al., 2022).

Although great progress has been made in elucidating the critical roles of miRNAs in the initiation and progression of FSGS (Table 1), the impact of miRNAs on glomerular structure as well as podocyte foot process effacement have yet to be investigated in detail. Moreover, currently it is still difficult to figure out the exact molecular mechanisms involved in the regulation of urinary and plasma miRNAs expression levels under different states of FSGS. Alternatively, a better understanding of what mediates the podocytes injury in FSGS will likely lead to novel therapeutic strategies. Given the complexity of miRNA regulation, more in-depth studies are necessary to fully understand their contribution to FSGS.

**TABLE 1:** MiRNAs in focal segmental glomerulosclerosis.

MiRNA	Dysregulation	Target	*In vitro* model	*In vivo* model	Effects	Reference
miR-193a	Up	WT1	Podocytes	FSGS patients FSGS mice	Promotes podocyte dysfunction	Gebeshuber et al. (2013)
miR-193a	Up	**—**	Podocytes	FSGS patients	As a diagnostic marker	Wang et al. (2021)
miR-490 miR-196a miR-30a-5p	Up	**—**	**—**	FSGS patients	Positively associated with disease activity	Zhang et al. (2014)
miR-186	Up	**—**	**—**	FSGS patients	As a specific biomarker	Zhang et al. (2015)
miR-30s	Down	Notch1 and p53	Podocytes	FSGS patients FSGS rats	Loss of miR-30s facilitates podocyte injury	Wu et al. (2014)
miR-30s	Down	TRPC6, PPP3CA, PPP3CB, PPP3R1, NFATC3	Podocytes	FSGS patients FSGS rats	As essential regulators of calcium/calcineurin signaling	Wu et al. (2015)
miR-135a	Up	TRPC1	Podocytes	FSGS patients FSGS mice	Promotes podocyte injury and apoptosis	Yang et al. (2017)
miR-155	Up	**—**	**—**	FSGS patients	As a diagnostic and prognostic maker	Ramezani et al. (2015)
miR-663 miR-1915	Down	**—**	**—**	FSGS patients	As a diagnostic and prognostic maker	Ramezani et al. (2015)
miR-206	Up	WT1	Podocytes	FSGS mice	Promotes podocyte injury	Guo et al. (2016)
miR-150	Up	SOCS1	Podocytes	FSGS patients FSGS mice	LNA-anti-miR-150 attenuates podocyte injury	Qi et al. (2020)
miR-106a	Down	CXCL14	Podocytes	FSGS patients	Suppresses podocyte apoptosis	Xiao et al. (2018)
miR-146b-5p	Up	TRAF6	**—**	FSGS patients	As a new type of biomarker	Williams et al. (2022)

### miRNAs in Minimal Change Disease

Minimal change disease (MCD) is one of the main causes of nephrotic syndrome, which is typically defined as lacking significant visible glomerular structure alterations by light microscopy and the widespread effacement of podocyte foot processes on electron microscopy without electron-dense deposits (Floege and Amann, 2016). Although the exact etiology of MCD remains unclear, glomerular permeability factors are considered as important contributors to the pathogenesis of MCD (Clement et al., 2011). Current treatment of MCD predominantly relies on corticosteroids (Maas et al., 2016). However, steroid-sensitive individuals frequently develop recrudesce or steroid resistance, which eventually results in a part of patients requiring second-line steroid-sparing immunosuppression (Vivarelli et al., 2017). Steroid-resistance occurs in approximately 10%–30% of adult cases, which occurs more frequently and earlier in children (Waldman et al., 2007). Recently, several newer agents, such as rituximab, have been utilized in adult patients with MCD aiming to decrease the risk of adverse effects of steroid therapies. However, due to the extremely high cost, rituximab currently cannot replace steroid as a first-line treatment for MCD (Munyentwali et al., 2013). Moreover, in the pediatric setting, renal biopsy is usually not performed unless steroid-resistance is observed (Vivarelli et al., 2017). Hence, there is an urgent need for developing alternative strategies to treat MCD as well as identifying novel non-invasive methods to diagnose MCD.

Recent evidence has revealed that dysregulation of certain miRNAs is linked to MCD progression and clinical outcome, suggesting they might be considered as potential diagnostic markers and therapeutic targets for MCD. To date, numerous studies have demonstrated the critical role of miRNAs in FSGS progression. In this regard, published data on MCD is relatively scarce. One recent study has reported that exogenous overexpression of miR-499 could ameliorate MCD symptoms as well as attenuate the foot-process effacement of podocytes in PAN-induced MCD mouse model (Zhang et al., 2018). Mechanistical studies showed that miR-499 may exert a protective effect on podocytes by suppressing the expression of the catalytic calcineurin isoforms α (CnAα) and β (CnAβ), leading to a decreased activity of calcineurin signaling in podocytes. Zheng et al. identified that miR-27b aggravated PAN-induced podocyte dysfunction in a primary podocyte model through targeting inhibition of adenosine receptor 2B (Adora2b), an intracellular pro-survival protein (Zheng et al., 2018). Lu et al. proposed that miR-150 had the potential to differentiate MCD from other nephropathy subtypes (Lu et al., 2015). In addition, a growing body of evidence has indicated that dysregulation of certain miRNAs in body fluid of patients with MCD may serve as diagnostic markers and prognostic indicators, mainly including plasma levels of miR-192 (Cai et al., 2013), miR-30b, miR-30c, miR-34b, and miR-34c (Ramezani et al., 2015) and urine level of miR-1225-5p (Ramezani et al., 2015).

Although many miRNAs have been identified to be dysregulated in MCD (Table 2), the precise mechanism of action of these miRNAs in MCD pathology remains unclear due to the lack of direct *in vivo* experimental evidence. Furthermore, since researchers have proposed that MCD and FSGS are in fact different histological manifestations of the same disease processes, those miRNAs that were found to play a key role in FSGS may also be involved in the pathogenesis of MCD, which deserves to be further investigated.

**TABLE 2:** MiRNAs in minimal change disease.

MiRNA	Dysregulation	Target	*In vitro* model	*In vivo* model	Effects	Reference
miR-499	Down	CnAα, CnAβ	Podocytes	MCD mice	Protects podocytes from cytoskeletal damage	Zhang et al. (2018)
miR-27b	Down	Adora2b	Podocytes	**—**	Enhances PAN-induced podocytes death	Zheng et al. (2018)
miR-150	Down	**—**	**—**	MCD patients	As a potential typing indictor	Lu et al. (2015)
miR-192	Up	**—**	**—**	MCD patients	As a diagnostic and prognostic maker	Cai et al. (2013)
miR-30b/c, miR-34b/c	Up	**—**	**—**	MCD patients	As a diagnostic maker	Ramezani et al. (2015)
miR-1225-5p	Up	**—**	**—**	MCD patients	As a diagnostic maker	Ramezani et al. (2015)

### MiRNAs in Membranous Nephropathy

Membranous nephropathy (MN) is defined as a complex pathological disorder of the glomeruli that occurs sporadically in all age groups (Couser, 2017). The deposition of immune complexes and the formation of membrane attack complexes contribute to the structural disturbances in podocytes, which ultimately lead to the development of massive proteinuria (Ronco et al., 2021). Clinically, MN often presents as nephrotic syndrome with variable outcomes, among which one-third of patients remit spontaneously, another third display variable degrees of persistent proteinuria without renal function exacerbation, while the remaining third develop to ESKD (Ronco et al., 2021). Cyclophosphamide-based therapy protocols have long been the standard therapy because they have been shown to prevent the occurrence of renal failure, but they put patients at a higher risk of developing cancer (Cattran and Brenchley, 2017). Besides, treatment plans with CD20-targeting agents are well tolerated, but patients achieve durable clinical remissions at low rates, and strong evidence of their effectiveness in preventing renal disease progression is still lacking (Ruggenenti et al., 2017; Ronco et al., 2021). Accordingly, developing new antigen-specific immunotherapies as well as identifying novel diagnostic and therapeutic targets for MN are of great importance for the development of effective therapeutic strategies to battle this disease.

With the continuous advancement of high-throughput sequencing technology and calculation methods, hundreds of dysregulated miRNAs have been identified and are proposed to potentially play significant roles in the pathogenesis of MN (Table 3). Nevertheless, only few miRNAs have been confirmed to be involved in MN. For instance, Sha et al. analyzed renal tissues from MN patients and found that miR-186 was significantly decreased in MN (Sha et al., 2015). In addition, they demonstrated that Ang II treatment significantly down-regulated the level of miR-186 in cultured podocytes, while ectopic expression of miR-186 attenuated Ang II-induced podocytes apoptosis. The expression levels of miR-217 were found to be consistently decreased in MN tissue and plasma by Li and her colleagues (Li et al., 2017). *In vitro* studies proposed that miR-217 silencing induced podocytes apoptosis through targeting tumor necrosis factor superfamily member 11 (TNFSF11). However, whether miR-217 could regulate TNFSF11 expression *in vivo*, and whether such relation between them could make a contribution to the pathology of MN, still require further investigation. Nonetheless, they proposed that absolutely quantifying plasma miR-217 could be an advantageous diagnostic biomarker for MN. Besides, another research also identified that miR-130a-5p was downregulated in the renal biopsy specimens from MN patients, which could attenuate Ang II induced-podocyte apoptosis through modulating PLA2R expression (Liu et al., 2018), however, this study also lacked *in vivo* experiments. Apart from these, some miRNAs, not yet experimentally confirmed, were proposed as potential diagnostic biomarkers for MN, including miR-98, miR-375, miR-7-5p, miR-615-3p, miR-577 (Chen et al., 2014), let-7a-5p, let-7c-5 (Barbagallo et al., 2019), miR-195-5p, miR-192-3p, miR-328-5p (Zhou et al., 2019), miR-106a and miR-19b (Wu et al., 2021), indicating that the exact role of miRNAs in the occurrence and the progression of MN is far less understood and remains an enigma.

**TABLE 3:** MiRNAs in membranous nephropathy.

MiRNA	Dysregulation	Target	*In vitro* model	*In vivo* model	Effects	Reference
miR-186	Down	P2X7	Podocytes	MN patients	Antiapoptotic effect of podocytes	Sha et al. (2015)
miR-217	Down	TNFSF11	Podocytes	MN patients	As a useful diagnostic biomarker	Li et al. (2017)
miR-130a-5p	Down	PLA2R	Podocytes	MN patients MN mice	Prevents angiotensin II-induced podocyte apoptosis	Liu et al. (2018)
miR-98 miR-375	Up	**—**	**—**	MN patients	As novel biomarkers for the diagnosis and treatment	Chen et al. (2014)
miR-7-5p miR-615-3p miR-577	Down	**—**	**—**	MN patients	As novel biomarkers for the diagnosis and treatment	Chen et al. (2014)
let-7a-5p let-7c-5p	Up	IL6 MYC	**—**	MN patients	As potential diagnostic biomarkers	Barbagallo et al. (2019)
miR-195-5p	Up	PPM1A	**—**	MN patients	As a potential biomarker	Zhou et al. (2019)
miR-192-3p	Up	RAB1A	**—**	MN patients	As a potential biomarker	Zhou et al. (2019)
miR-328-5p	Down	BRSK1	**—**	MN patients	As a potential biomarker	Zhou et al. (2019)
miR-106a miR-19b	Down	PTEN	**—**	MN patients	As new biomarkers for the diagnosis of MN	Wu et al. (2021)

Collectively, based on these current reports, targeting inhibition or ectopic expression of certain miRNAs in podocytes might yield new strategies for MN diagnosis, prevention, and therapy.

### MiRNAs in Diabetic Kidney Disease

Diabetic kidney disease (DKD) is a major microvascular complication of diabetes mellitus and the leading cause of chronic kidney disease (CKD) and ESKD worldwide, which occurs in approximately one-third of type 1 diabetes mellitus (T1DM) patients and 40% of type 2 diabetes mellitus (T2DM) patients (Alicic et al., 2017). The global surge in DKD prevalence parallels the dramatic increase in the incidence of diabetes worldwide (de Boer et al., 2011). Progressive increase in proteinuria and progressive deterioration of renal function are major clinical manifestations of DKD (Doshi and Friedman, 2017). Despite current strategies for DKD management, mainly including lifestyle modification, intensive control of glycemic, blood pressure and lipid as well as albuminuria-reducing, have meaningfully improved outcomes for diabetes complications, DKD still poses a major risk factor for the development of ESKD (Alicic et al., 2017). Multiple lines of evidence have demonstrated that podocyte injury or loss plays a critical role in the development and progression of DKD, which ultimately leading to proteinuria and further renal damage (Mathieson, 2011; Reidy et al., 2014). Therefore, identifying the key molecules that may be involved in podocyte injury will provide new clues in developing novel diagnostic, therapeutic and prevention strategies for DKD.

Growing body of evidence suggests that dysregulation various of miRNAs may serve not only as diagnostic biomarkers but also as therapeutic targets for several malignant tumors (Beg et al., 2017; van Zandwijk et al., 2017). Furthermore, in recent years, with the deepening of researches, the roles of miRNAs in the pathogenesis of podocyte injury in DKD have received more attention. Many previous studies have systematically elucidated that the ectopic expression or knock down/out of indicated miRNAs could exert a significant effect on high glucose (HG) induced podocytes injury *in vitro*, as well as in rodent models of DKD. For example, ectopic expression of miRNA-23b in diabetic kidneys attenuated diabetes-induced podocyte injury, reduced proteinuria as well as effectively mitigated DKD progression in diabetic mice, indicating a protective role of miR-23b in DKD podocyte injury (Zhao et al., 2016). Another study by the same research group reported that miR-25 expression levels were significantly decreased in the sera of diabetic patients as well as in kidney tissues from diabetic mice (Liu et al., 2017). Further *in vivo* studies indicated that overexpression of miRNA-25 via intravenous injection of an miR-25 mimic was shown to ameliorate podocyte injury in diabetic mice. Mechanistic studies revealed that miRNA-25 exerted its protective role in DKD mainly through targeting inhibition of cell division cycle 42 (CDC42) expression, a downstream effector of Ras that can lead to congenital nephrotic syndrome and glomerulosclerosis. A more recent study also suggested the protective role of miRNA against podocyte injury in DKD. In this study, miR-10a and miR-10b were identified to be predominantly expressed in the kidney and significantly downregulated in podocytes under diabetic conditions, which acted as endogenous inhibitors of the NLRP3 inflammasome in DKD, thereby protecting podocytes against injury in DKD (Ding et al., 2021). Besides, other studies have also reported the protective role of miRNAs in against podocyte injury DKD, such as miR-146a (Lee et al., 2017), miR-29a (Lin et al., 2014), and miR-93 (Badal et al., 2016), which may act as potential therapeutic targets for DKD. While, Zhou et al. have demonstrated that up-regulated miR-27a exacerbated HG-induced podocytes injury *in vitro* and contributed to unfavorable renal function and increased podocyte injury in diabetic rats *in vivo* (Zhou et al., 2017). In addition, Kölling and colleagues suggested that therapeutic miR-21 silencing could ameliorate DKD in mice models (Kölling et al., 2017). Apart from these, other miRNAs also played detrimental roles in diabetes-induced podocytes injury, like miR-29c (Long et al., 2011), miR-182-5p (Ming et al., 2019), miR-20b (Wang et al., 2017), miR-503 (Zha et al., 2019), miR-193a (Mishra et al., 2018) and so on. In a word, dysregulation of many miRNAs indeed plays various harmful roles in the initiation and progression of podocyte injury in DKD (Table 4).

**TABLE 4:** MiRNAs in diabetic kidney disease.

MiRNA	Dysregulation	Target	*In vitro* model	*In vivo* model	Effects	Reference
miR-23b	Down	G3BP2	Podocytes	DKD patients DKD mice	Exerts protective effects against podocyte injury	Zhao et al. (2016)
miR-25	Down	CDC42	Podocytes	DKD patients DKD mice	Shows protective effects against podocyte injury	Liu et al. (2017)
miR-10a/b	Down	NLRP3	Podocytes	DKD patients DKD mice	Negatively regulates inflammation in diabetic kidney	Ding et al. (2021)
miR-146a	Down	Notch1 ErbB4	Podocytes	DKD mice	As a biomarker for disease progression	Lee et al. (2017)
miR-29a	Down	HDAC4	Podocytes	DKD mice	Ameliorates diabetes-induced podocyte injury	Lin et al. (2014)
miR-93	Down	Msk2	Podocytes	DKD patients DKD mice	Attenuates podocyte injury	Badal et al. (2016)
miR-27a	Up	PPARγ	Podocytes	DKD patients DKD rats	Promotes podocyte injury	Zhou et al. (2017)
miR-21	Up	PTEN	Podocytes	DKD patients DKD mice	Promotes podocyte dysfunction	Kölling et al. (2017)
miR-29c	Up	SPRY1	Podocytes	DKD mice	As a novel therapeutic target in diabetic nephropathy	Long et al. (2011)
miR-182-5p	Up	CD2AP	Podocytes	DKD patients	Induces podocyte apoptosis	Ming et al. (2019)
miR-20b	Up	SIRT7	Podocytes	**—**	Contribute to HG-induced podocytes apoptosis	Wang et al. (2017)
miR-503	Up	E2F3	Podocytes	DKD rats	Contributes to podocyte injury	Zha et al. (2019)
miR-193a	Up	APOL1	Podocytes	**—**	Prevents podocytes dedifferentiation in HG condition	Mishra et al. (2018)

These lines of evidence suggest that miRNAs have dual roles in DKD-induced podocyte injury, targeting inhibition or ectopic expression of indicated miRNAs in podocytes would have protective effects against podocyte injury in DKD, thus deserving further investigation.

### MiRNAs in IgA Nephropathy

IgA nephropathy (IgAN) is the most common primary glomerulonephritis worldwide (Roberts, 2014; Rodrigues et al., 2017). Patients with IgAN can present with a wide range of symptoms, from asymptomatic microscopic hematuria to rapidly progressive glomerulonephritis (Roberts, 2014; Rodrigues et al., 2017). Currently, there is still no specific treatment for IgAN and patients are managed with the aim of controlling blood pressure and maintaining renal function. The pathology of IgAN is mainly characterized by the deposition of pathogenetic polymeric IgA_1_-IgG immune complexes in the glomerular mesangium, proliferation of mesangial cells, increased synthesis of extracellular matrix and infiltration of many types of immune cells, including macrophages, monocytes and T cells (Lai et al., 2008; Lai et al., 2016). In addition, emerging evidence indicates that podocyte injury is singularly important in the pathogenesis of IgAN and has been considered as a key mechanism leading to the disease progression (Hill et al., 2011; Lai et al., 2016). Nevertheless, the precise molecular mechanisms underlying podocyte injury in IgAN have not been fully understood. Thus, it is urgent to identify novel key molecules underlying podocyte injury in IgAN that can potentially serve as early diagnostic biomarkers and/or therapeutic targets for IgAN.

Over the past decade, we have witnessed a flourishing of studies aimed at exploring the biological functions of miRNAs in the occurrence and development of IgAN (Szeto and Li, 2014). Hundreds of dysregulated miRNAs have been identified in IgAN, some of which have been proposed to play significant roles in the pathogenesis of IgAN. For example, Dai and his colleagues performed the first genome-wide analysis of miRNAs expression profiling in kidney biopsy samples from 11 patients with IgAN and 3 normal controls and found that the expression levels of miR-200c, miR-141, miR-205 and miR-192 were associated with disease severity and progression in IgAN patients (Dai et al., 2008). In another study, they further investigated miRNA expression in renal biopsy samples from six patients with IgAN and normal renal cortex samples from six patients with renal cancer by high-throughput sequencing technology and identified 11 upregulated miRNAs and 74 downregulated miRNAs in IgAN. Further bioinformatic analysis indicated that these dysregulated miRNAs were mainly involved in the regulation of the macro molecular metabolism, the nitrogen compound metabolic process and biosynthetic process (Tan et al., 2013). Although neither study further explored the exact roles of these dysregulated miRNAs in IgAN, they provided new directions for investigating the pathogenesis of IgAN. Another study found that miR-21 expression level was up-regulated remarkably in glomerular tissues of patients with IgAN compared to that of the healthy control people (Bao et al., 2014b). Further studies revealed that mesangial-derived cytokines could up-regulate miR-21 in podocytes and inhibition of miR-21 prevented fibrogenic activation in podocytes. Interestingly, the relationship between miR-21 and podocytopathy is inspired by the clinical study conducted by Kong et al., which had found that the actual level of urinary albumin excretion correlated with the urinary level of miR-21 (Kong et al., 2012), indicating miR-21 may be involved in the pathogenetic mechanisms linking albuminuria in IgAN. Besides, Guo et al. indicated that miR-200b/c/429 cluster alleviated inflammation in IgAN by targeting TNF-like weak inducer of apoptosis (TWEAK) (Guo and Liao, 2017). And Osamu et al. suggested that glomerular miR-26a expression decreased significantly in both IgAN mice and patients, which was closely linked to the progression of podocyte injury in IgAN (Ichii et al., 2014). In addition, other studies have also reported the involvement of some other dysregulated miRNAs in IgAN, such as miR-223 (Bao et al., 2014a), miR-590-3p (Zhai et al., 2019), miR-133a/b (Jin et al., 2018), miR-320 (Li et al., 2018), miR-100-3p, miR-877-3p (Liang et al., 2016), miR-23b (Li H. et al., 2021), miR-214-3p (Li Y. et al., 2021), and miR-150-5p (Pawluczyk et al., 2021). IgAN appears to be a systemic disease in which the kidneys are damaged as innocent bystanders (Wyatt and Julian, 2013). Serino et al., for the first time, analyzed their global miRNA expression profile in peripheral blood mononuclear cells (PBMCs) of seven IgAN patients and seven healthy participants and revealed a novel pathophysiological mechanism whereby upregulation of miR-148b contributed to the aberrant IgA_1_ glycosylation through inhibiting core 1, β1, 3-galactosyltransferase 1 (C1GALT1) mRNA expression, providing a potential pharmacologic target for IgAN (Serino et al., 2012). Intriguingly, this result was further confirmed in a recent study that miR-148b expression level was significantly up-regulated in tissues from IgAN patients and positively correlated with eGFR (Wen et al., 2018). Apart from these, many circulating or urine miRNAs, not yet experimentally confirmed, were proposed as potential diagnostic biomarkers for IgAN, like miR-146a, miR-155 (Wang et al., 2011), miR-148b and let-7b (Serino et al., 2016; Kouri et al., 2021), indicating that these circulating and urine miRNAs may have a good potential for diagnosing IgAN and they deserve to be fully exploited in the future.

Despite the limited number of available studies, some interesting miRNAs have been identified as potentially relevant to the pathogenesis of IgAN (Table 5). Although the above-mentioned studies shed new light on the pathogenesis of IgAN, these studies all included small sample sizes and their results had limited adjustment for multiple potential clinical confounders. Furthermore, there is currently no accepted standard protocol for functional assessment of identified candidate miRNAs. Therefore, further research is urgently needed to address those shortages.

**TABLE 5:** MiRNAs in IgA nephropathy.

MiRNA	Dysregulation	Target	*In vitro* model	*In vivo* model	Effects	Reference
miR-21	Up	PTEN	Podocytes	IgAN patients	Inhibition of miR-21 prevented fibrogenic activation	Bao et al. (2014b)
miR-200b miR-200c miR-429	Down	TWEAK	Podocytes	IgAN patients	Alleviates inflammation, serve as promising therapeutic target	Guo and Liao, (2017)
miR-26a	Down	**—**	Podocytes	IgAN patients IgAN mice	Regulates podocyte differentiation and cytoskeletal integrity	Ichii et al. (2014)
miR-223	Down	Importin α4 and α5	GEnCs	IgAN patients	Provide a noninvasive method for evaluating the severity of IgAN	Bao et al. (2014a)
miR-590-3p	Up	HMGB2	PBMCs	IgAN patients	May contributes to the severity of IgAN	Zhai et al. (2019)
miR-133a miR-133b	Up	FOXP3	PBMCs	IgAN patients	Inhibits Treg differentiation in IgAN	Jin et al. (2018)
miR-148b	Up	C1GALT1	PBMCs	IgAN patients	Provides novel therapeutic approaches to IgAN	Serino et al. (2012)
miR-320	Up	PTEN	Peripheral B cells	IgAN patients	Promotes the B cell proliferation	Li et al. (2018)
miR-100-3p miR-877-3p	Down	IL-8 IL-1β	Mesangial cells	IgAN patients	Regulate overproduction of IL-8 and IL-1β in mesangial cells	Liang et al. (2016)
miR-23b	Down	Gremlin 2	Mesangial cells	IgAN patients IgAN mice	Offer a novel therapeutic target for the treatment of IgAN	Li et al. (2021a)
miR-214-3p	Up	PTEN	Mesangial cells	IgAN patients IgAN mice	Accelerates Mesangial cells proliferation	Li et al. (2021b)
miR-150-5p	Up	**—**	**—**	IgAN patients	As a potential mediator and marker of disease progression	Pawluczyk et al. (2021)
miR-148b	Up	MEGALIN	LLC-PK1	IgAN patients	May affect renal uptake and metabolism of essential substances	Wen et al. (2018)
miR-146a miR-155	Up	**—**	**—**	IgAN patients	Suggests an immunoregulatory role	Wang et al. (2011)
miR-148b let-7b	Up	**—**	**—**	IgAN patients	Appears to be novel noninvasive biomarkers	Serino et al. (2016), Kouri et al. (2021)

**FIGURE 2:**
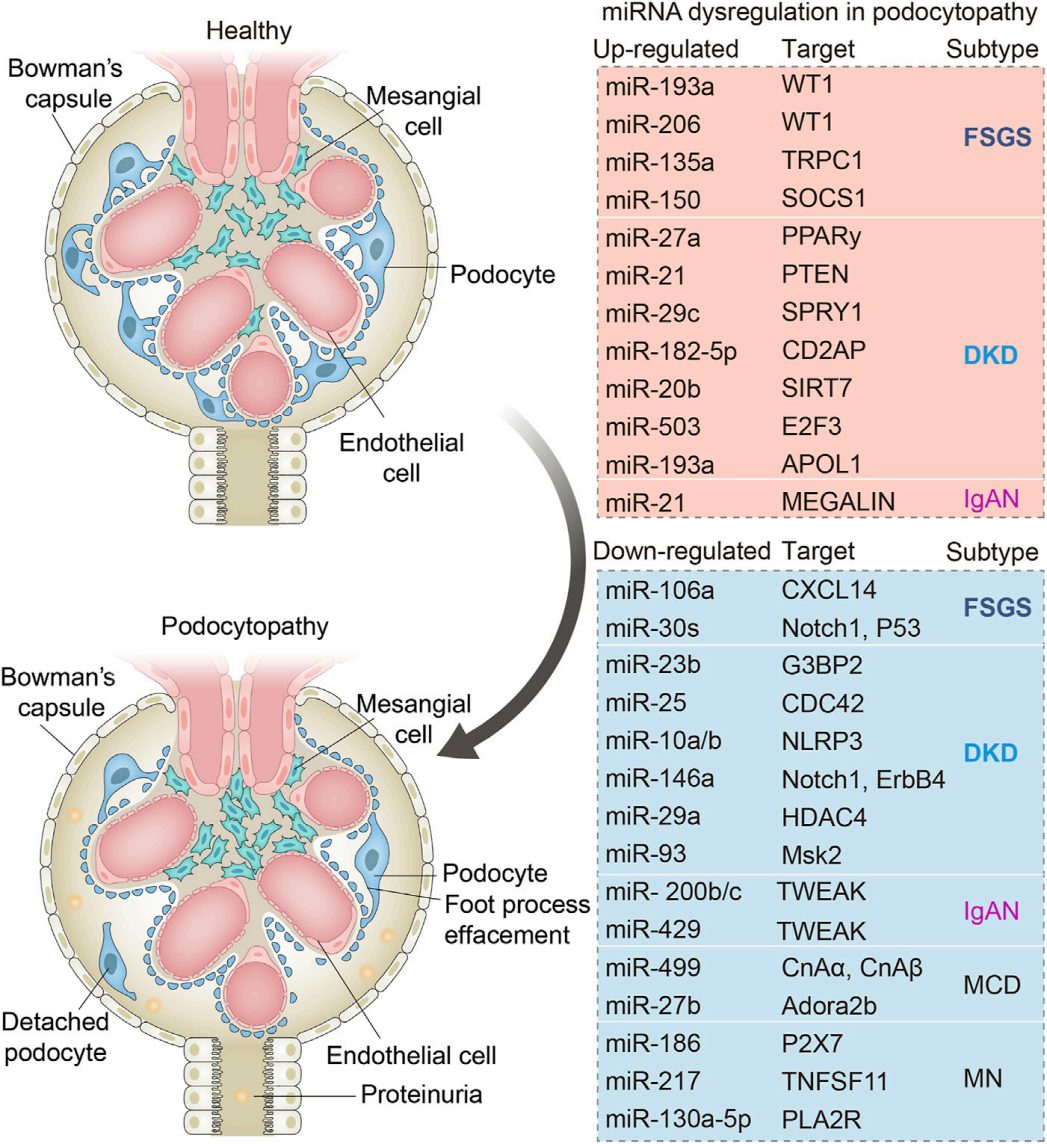
Schematic diagram showing the main dysregulated miRNAs and their corresponding downstream targets in various podocytopathies. Changes in miRNAs in podocytes occurring in focal segmental glomerulosclerosis (FSGS), minimal change disease (MCD), membranous nephropathy (MN), diabetic kidney diseases (DKD), and IgA nephropathy (IgAN). Dysregulation of indicated miRNAs contributes to podocyte injuries including podocyte foot process effacement and podocyte loss due to cell death or detachment from the glomerular basement membrane.

Collectively, the above studies indicated that miRNAs may have played critical regulatory roles in the processes of podocytopathies (Figure 2), which may serve as promising diagnostic biomarkers as well as therapeutic targets clinically.

### Potential Therapeutic Applications of MiRNAs

Mature miRNAs have unique characteristics, including short sequences as well as high homology across multiple vertebrate species, which make them potentially suitable as therapeutic targets for the treatment of various diseases. Currently, targeted manipulation of miRNAs in kidney can be achieved through exogenous delivery of specific miRNA inhibitors to block miRNA function or synthetic miRNA mimics to restore the expression level of a particular miRNA. However, no miRNAs are in clinical trials for podocytopathies at the moment. Whereas, to date, there are already some miRNA-based trials that have entered phase I or phase II clinical trials, showing significant clinical promise (Mellis and Caporali, 2018). This includes miR-16-based miRNA mimic for malignant pleural mesothelioma (van Zandwijk et al., 2017), miR-34a mimics in patients with advanced solid tumors (Beg et al., 2017; Hong et al., 2020), anti-miR-103 and anti-miR-107 for the treatment of type 2 diabetes and obesity (Trajkovski et al., 2011), miR-29a-3p mimics for scleroderma (Maurer et al., 2010), anti-miR-155 for cutaneous T-cell lymphoma and mycosis fungoides (Babar et al., 2012), anti-miR-122 for chronic hepatitis C virus (HCV) infection (Janssen et al., 2013), and anti-miR-21 for Alport syndrome (Gomez et al., 2015).

Many *in vivo* studies have focused on the therapeutic potential of manipulation of miRNAs in podocytopathies and promising therapeutic results in halting podocyte injury and ameliorating proteinuria have been achieved by knocking down miR-193a (Gebeshuber et al., 2013; Mishra et al., 2018), miR-150 (Qi et al., 2020), miR-27a (Zhou et al., 2017), and miR-21 (Kölling et al., 2017) or ectopic expressing miR-30s (Wu et al., 2014; Wu et al., 2015), miR-499 (Zhang et al., 2018), miR-27b (Zheng et al., 2018), miR-93 (Badal et al., 2016), miR-23b (Zhao et al., 2016), miR-25 (Liu et al., 2017), miR-10 (Ding et al., 2021) and miR-29a (Lin et al., 2014). Currently, efficient kidney *in vivo* transfection has been successfully achieved through intraperitoneal, intravenous or subcutaneous injection of indicated miRNA mimics or inhibitors (Trionfini et al., 2015; Henique et al., 2017). However, despite promising therapeutic applications, several obstacles must be conquered before miRNA-based therapies for podocytopathies can ultimately be translated into clinical practice, mainly including developing efficient methods to specific delivery of miRNA mimics or inhibitors to podocytes as well as avoiding the potential toxicity and adverse effects in other tissues and organs. Fortunately, so far, several strategies have been developed to partially overcome these limitations, such as using plasmids expressing indicated miRNAs or short-hairpin RNAs containing podocyte-specific promoters. In addition, the current standing diagnosis of podocytopathies predominately relays on kidney biopsy (Kopp et al., 2020), hence, there is an urgent need to develop novel non-invasive diagnostic methods for the detection and monitoring of such diseases. Emerging evidence suggests that many circulating or urine miRNAs are useful indicators of podocytopathies, such as miR-193a (Wang et al., 2021), miR-186 (Zhang et al., 2015), miR-192 (Cai et al., 2013), miR-217 (Li et al., 2017), and miR-146a (Lee et al., 2017). Therefore, measuring indicated miRNAs in the urine or plasma as a potential mechanism for monitoring accelerated podocyte loss non-invasively holds good potential for clinical application, which warrants further validation.

## Conclusion

In this review, we summarized current literature related to the pathogenic role of miRNA dysregulation in the pathogenesis of podocytopathies. We majorly focused on narrating the roles and values of these dysregulated miRNAs in the occurrence, progression, clinical diagnosis, treatment and prognosis of podocytopathies, which may provide theory and clinical basis for the future clinical treatment and research of this rare disease. Over the past few decades our knowledge towards the critical role of podocytes in the onset and progression of proteinuric kidney disease has achieved tremendous progress. The importance of miRNAs in the field of podocytopathies is increasingly recognized as they allow researchers to gain a deeper understanding of podocytopathies pathways as well as provide a rationale for the development of novel diagnostic and possibly therapeutic strategies. Moreover, the strategies of miRNA-based therapy that either restores or abolishes miRNA expression and activity are very attractive, especially now that the several miRNA-targeted therapeutics have reached clinical development. By now, targeted manipulation of miRNA expression as an effective antiproteinuric therapy has been revealed only in experimental models of podocytopathies because many safety concerns have not been settled, from the delivery methods to the potential toxicity and adverse effects in other tissues and organs. Furthermore, due to the highly pleiotropic nature of miRNAs, it is unlikely that a single miRNA will be able to accurately diagnose and predict a certain disease. Given the involvement of many different miRNAs in podocytopathies, we firmly believe that a panel of miRNAs would potentially be more useful than a single miRNA as a biomarker. Collectively, further research in this area will continue to identify new therapeutic targets as well as sensitive and specific diagnostic biomarkers for podocytopathies.

## References

[B1] Agbu P. Carthew R. W. (2021). MicroRNA-mediated Regulation of Glucose and Lipid Metabolism. Nat. Rev. Mol. Cell. Biol. 22 (6), 425–438. 10.1038/s41580-021-00354-w 33772227 PMC8853826

[B2] Alicic R. Z. Rooney M. T. Tuttle K. R. (2017). Diabetic Kidney Disease. Clin. J. Am. Soc. Nephrol. 12 (12), 2032–2045. 10.2215/cjn.11491116 28522654 PMC5718284

[B3] Arif E. Solanki A. K. Srivastava P. Rahman B. Fitzgibbon W. R. Deng P. (2019). Mitochondrial Biogenesis Induced by the β2-adrenergic Receptor Agonist Formoterol Accelerates Podocyte Recovery from Glomerular Injury. Kidney Int. 96 (3), 656–673. 10.1016/j.kint.2019.03.023 31262488 PMC6708766

[B4] Ayalon R. Beck L. H. Jr. (2015). Membranous Nephropathy: Not Just a Disease for Adults. Pediatr. Nephrol. 30 (1), 31–39. 10.1007/s00467-013-2717-z 24375012 PMC4074564

[B5] Babar I. A. Cheng C. J. Booth C. J. Liang X. Weidhaas J. B. Saltzman W. M. (2012). Nanoparticle-based Therapy in an *In Vivo* microRNA-155 (miR-155)-dependent Mouse Model of Lymphoma. Proc. Natl. Acad. Sci. U.S.A. 109 (26), E1695–E1704. 10.1073/pnas.1201516109 22685206 PMC3387084

[B6] Badal S. S. Wang Y. Long J. Corcoran D. L. Chang B. H. Truong L. D. (2016). miR-93 Regulates Msk2-Mediated Chromatin Remodelling in Diabetic Nephropathy. Nat. Commun. 7, 12076. 10.1038/ncomms12076 27350436 PMC4931323

[B7] Bao H. Chen H. Zhu X. Zhang M. Yao G. Yu Y. (2014a). MiR-223 Downregulation Promotes Glomerular Endothelial Cell Activation by Upregulating Importin α4 and α5 in IgA Nephropathy. Kidney Int. 85 (3), 624–635. 10.1038/ki.2013.469 24284509

[B8] Bao H. Hu S. Zhang C. Shi S. Qin W. Zeng C. (2014b). Inhibition of miRNA-21 Prevents Fibrogenic Activation in Podocytes and Tubular Cells in IgA Nephropathy. Biochem. Biophysical Res. Commun. 444 (4), 455–460. 10.1016/j.bbrc.2014.01.065 24468088

[B9] Barbagallo C. Passanisi R. Mirabella F. Cirnigliaro M. Costanzo A. Lauretta G. (2019). Upregulated microRNAs in Membranous Glomerulonephropathy Are Associated with Significant Downregulation of IL6 and MYC mRNAs. J. Cell. Physiology 234 (8), 12625–12636. 10.1002/jcp.27851 PMC13484095 30515781

[B10] Beg M. S. Brenner A. J. Sachdev J. Borad M. Kang Y.-K. Stoudemire J. (2017). Phase I Study of MRX34, a Liposomal miR-34a Mimic, Administered Twice Weekly in Patients with Advanced Solid Tumors. Invest. New Drugs 35 (2), 180–188. 10.1007/s10637-016-0407-y 27917453 PMC5893501

[B11] Blanco-Domínguez R. Sánchez-Díaz R. de la Fuente H. Jiménez-Borreguero L. J. Matesanz-Marín A. Relaño M. (2021). A Novel Circulating MicroRNA for the Detection of Acute Myocarditis. N. Engl. J. Med. 384 (21), 2014–2027. 10.1056/NEJMoa2003608 34042389 PMC8258773

[B12] Borchert G. M. Lanier W. Davidson B. L. (2006). RNA Polymerase III Transcribes Human microRNAs. Nat. Struct. Mol. Biol. 13 (12), 1097–1101. 10.1038/nsmb1167 17099701

[B13] Cai X. Xia Z. Zhang C. Luo Y. Gao Y. Fan Z. (2013). Serum microRNAs Levels in Primary Focal Segmental Glomerulosclerosis. Pediatr. Nephrol. 28 (9), 1797–1801. 10.1007/s00467-013-2434-7 23463342 PMC3722457

[B14] Cattran D. C. Brenchley P. E. (2017). Membranous Nephropathy: Integrating Basic Science into Improved Clinical Management. Kidney Int. 91 (3), 566–574. 10.1016/j.kint.2016.09.048 28065518

[B15] Chen W. Lin X. Huang J. Tan K. Chen Y. Peng W. (2014). Integrated Profiling of microRNA Expression in Membranous Nephropathy Using High-Throughput Sequencing Technology. Int. J. Mol. Med. 33 (1), 25–34. 10.3892/ijmm.2013.1554 24220188 PMC3868500

[B16] Clement L. C. Avila-Casado C. Macé C. Soria E. Bakker W. W. Kersten S. (2011). Podocyte-secreted Angiopoietin-Like-4 Mediates Proteinuria in Glucocorticoid-Sensitive Nephrotic Syndrome. Nat. Med. 17 (1), 117–122. 10.1038/nm.2261 21151138 PMC3021185

[B17] Cohen S. D. Kopp J. B. Kimmel P. L. (2017). Kidney Diseases Associated with Human Immunodeficiency Virus Infection. N. Engl. J. Med. 377 (24), 2363–2374. 10.1056/NEJMra1508467 29236630

[B18] Colucci M. Carsetti R. Rosado M. M. Cascioli S. Bruschi M. Candiano G. (2019). Atypical IgM on T Cells Predict Relapse and Steroid Dependence in Idiopathic Nephrotic Syndrome. Kidney Int. 96 (4), 971–982. 10.1016/j.kint.2019.04.006 31285081

[B19] Couser W. G. (2017). Primary Membranous Nephropathy. Clin. J. Am. Soc. Nephrol. 12 (6), 983–997. 10.2215/cjn.11761116 28550082 PMC5460716

[B20] Craici I. M. Wagner S. J. Weissgerber T. L. Grande J. P. Garovic V. D. (2014). Advances in the Pathophysiology of Pre-eclampsia and Related Podocyte Injury. Kidney Int. 86 (2), 275–285. 10.1038/ki.2014.17 24573315 PMC4117806

[B21] D'Agati V. D. Chagnac A. de Vries A. P. J. Levi M. Porrini E. Herman-Edelstein M. (2016). Obesity-related Glomerulopathy: Clinical and Pathologic Characteristics and Pathogenesis. Nat. Rev. Nephrol. 12 (8), 453–471. 10.1038/nrneph.2016.75 27263398

[B22] D'Agati V. D. Kaskel F. J. Falk R. J. (2011). Focal Segmental Glomerulosclerosis. N. Engl. J. Med. 365 (25), 2398–2411. 10.1056/NEJMra1106556 22187987

[B23] Dai Y. Sui W. Lan H. Yan Q. Huang H. Huang Y. (2008). Microarray Analysis of Micro-ribonucleic Acid Expression in Primary Immunoglobulin A Nephropathy. Saudi Med. J. 29 (10), 1388–1393. 10.1007/s00296-008-0758-6 18946560

[B24] de Boer I. H. Rue T. C. Hall Y. N. Heagerty P. J. Weiss N. S. Himmelfarb J. (2011). Temporal Trends in the Prevalence of Diabetic Kidney Disease in the United States. Jama 305 (24), 2532–2539. 10.1001/jama.2011.861 21693741 PMC3731378

[B25] Ding H. Li J. Li Y. Yang M. Nie S. Zhou M. (2021). MicroRNA-10 Negatively Regulates Inflammation in Diabetic Kidney via Targeting Activation of the NLRP3 Inflammasome. Mol. Ther. 29 (7), 2308–2320. 10.1016/j.ymthe.2021.03.012 33744467 PMC8261077

[B26] Doshi S. M. Friedman A. N. (2017). Diagnosis and Management of Type 2 Diabetic Kidney Disease. Clin. J. Am. Soc. Nephrol. 12 (8), 1366–1373. 10.2215/cjn.11111016 28280116 PMC5544517

[B27] Eng D. G. Sunseri M. W. Kaverina N. V. Roeder S. S. Pippin J. W. Shankland S. J. (2015). Glomerular Parietal Epithelial Cells Contribute to Adult Podocyte Regeneration in Experimental Focal Segmental Glomerulosclerosis. Kidney Int. 88 (5), 999–1012. 10.1038/ki.2015.152 25993321 PMC4654724

[B28] Fan Y. Chen H. Huang Z. Zheng H. Zhou J. (2020). Emerging Role of miRNAs in Renal Fibrosis. RNA Biol. 17 (1), 1–12. 10.1080/15476286.2019.1667215 31550975 PMC6948964

[B29] Filipowicz W. Bhattacharyya S. N. Sonenberg N. (2008). Mechanisms of Post-transcriptional Regulation by microRNAs: Are the Answers in Sight? Nat. Rev. Genet. 9 (2), 102–114. 10.1038/nrg2290 18197166

[B30] Floege J. Amann K. (2016). Primary Glomerulonephritides. Lancet 387 (10032), 2036–2048. 10.1016/s0140-6736(16)00272-5 26921911

[B31] Fogo A. B. (2015). Causes and Pathogenesis of Focal Segmental Glomerulosclerosis. Nat. Rev. Nephrol. 11 (2), 76–87. 10.1038/nrneph.2014.216 25447132 PMC4772430

[B32] Friedman R. C. Farh K. K.-H. Burge C. B. Bartel D. P. (2009). Most Mammalian mRNAs Are Conserved Targets of microRNAs. Genome Res. 19 (1), 92–105. 10.1101/gr.082701.108 18955434 PMC2612969

[B33] Fukuda A. Chowdhury M. A. Venkatareddy M. P. Wang S. Q. Nishizono R. Suzuki T. (2012). Growth-dependent Podocyte Failure Causes Glomerulosclerosis. J. Am. Soc. Nephrol. 23 (8), 1351–1363. 10.1681/asn.2012030271 22773827 PMC3402293

[B34] Gebeshuber C. A. Kornauth C. Dong L. Sierig R. Seibler J. Reiss M. (2013). Focal Segmental Glomerulosclerosis Is Induced by microRNA-193a and its Downregulation of WT1. Nat. Med. 19 (4), 481–487. 10.1038/nm.3142 23502960

[B35] Gomez I. G. MacKenna D. A. Johnson B. G. Kaimal V. Roach A. M. Ren S. (2015). Anti-microRNA-21 Oligonucleotides Prevent Alport Nephropathy Progression by Stimulating Metabolic Pathways. J. Clin. Invest. 125 (1), 141–156. 10.1172/jci75852 25415439 PMC4382246

[B36] Guo N. Guo J. Su D. (2016). MicroRNA-206 and its Down-Regulation of Wilms'Tumor-1 Dictate Podocyte Health in Adriamycin-Induced Nephropathy. Ren. Fail. 38 (6), 989–995. 10.3109/0886022x.2016.1165119 27056206

[B37] Guo Y. Liao Y. (2017). miR-200bc/429 Cluster Alleviates Inflammation in IgA Nephropathy by Targeting TWEAK/Fn14. Int. Immunopharmacol. 52, 150–155. 10.1016/j.intimp.2017.09.002 28910745

[B38] Hama T. Nakanishi K. Shima Y. Mukaiyama H. Togawa H. Tanaka R. (2012). Renal Biopsy Criterion in Children with Asymptomatic Constant Isolated Proteinuria. Nephrol. Dial. Transplant. 27 (8), 3186–3190. 10.1093/ndt/gfr750 22231035

[B39] Henique C. Bollée G. Loyer X. Grahammer F. Dhaun N. Camus M. (2017). Genetic and Pharmacological Inhibition of microRNA-92a Maintains Podocyte Cell Cycle Quiescence and Limits Crescentic Glomerulonephritis. Nat. Commun. 8 (1), 1829. 10.1038/s41467-017-01885-7 29184126 PMC5705755

[B40] Hill G. S. Karoui K. E. Karras A. Mandet C. Van Huyen J.-P. D. Nochy D. (2011). Focal Segmental Glomerulosclerosis Plays a Major Role in the Progression of IgA Nephropathy. I. Immunohistochemical Studies. Kidney Int. 79 (6), 635–642. 10.1038/ki.2010.466 21160460

[B41] Hong D. S. Kang Y.-K. Borad M. Sachdev J. Ejadi S. Lim H. Y. (2020). Phase 1 Study of MRX34, a Liposomal miR-34a Mimic, in Patients with Advanced Solid Tumours. Br. J. Cancer 122 (11), 1630–1637. 10.1038/s41416-020-0802-1 32238921 PMC7251107

[B42] Ichii O. Otsuka-Kanazawa S. Horino T. Kimura J. Nakamura T. Matsumoto M. (2014). Decreased miR-26a Expression Correlates with the Progression of Podocyte Injury in Autoimmune Glomerulonephritis. PLoS One 9 (10), e110383. 10.1371/journal.pone.0110383 25329154 PMC4201534

[B43] Iijima K. Sako M. Nozu K. Mori R. Tuchida N. Kamei K. (2014). Rituximab for Childhood-Onset, Complicated, Frequently Relapsing Nephrotic Syndrome or Steroid-dependent Nephrotic Syndrome: a Multicentre, Double-Blind, Randomised, Placebo-Controlled Trial. Lancet 384 (9950), 1273–1281. 10.1016/s0140-6736(14)60541-9 24965823

[B44] Janssen H. L. A. Reesink H. W. Lawitz E. J. Zeuzem S. Rodriguez-Torres M. Patel K. (2013). Treatment of HCV Infection by Targeting microRNA. N. Engl. J. Med. 368 (18), 1685–1694. 10.1056/NEJMoa1209026 23534542

[B45] Jin L.-W. Ye H.-Y. Xu X.-Y. Zheng Y. Chen Y. (2018). MiR-133a/133b Inhibits Treg Differentiation in IgA Nephropathy through Targeting FOXP3. Biomed. Pharmacother. 101, 195–200. 10.1016/j.biopha.2018.02.022 29494956

[B46] Kang Y. S. Li Y. Dai C. Kiss L. P. Wu C. Liu Y. (2010). Inhibition of Integrin-Linked Kinase Blocks Podocyte Epithelial-Mesenchymal Transition and Ameliorates Proteinuria. Kidney Int. 78 (4), 363–373. 10.1038/ki.2010.137 20505657 PMC3065782

[B47] Kato M. Arce L. Natarajan R. (2009). MicroRNAs and Their Role in Progressive Kidney Diseases. Clin. J. Am. Soc. Nephrol. 4 (7), 1255–1266. 10.2215/cjn.00520109 19581401 PMC2709511

[B48] Kim A. H. J. Chung J.-J. Akilesh S. Koziell A. Jain S. Hodgin J. B. (2017). B Cell-Derived IL-4 Acts on Podocytes to Induce Proteinuria and Foot Process Effacement. JCI Insight 2 (21), e81836. 10.1172/jci.insight.81836 PMC5752294 29093269

[B49] Kölling M. Kaucsar T. Schauerte C. Hübner A. Dettling A. Park J.-K. (2017). Therapeutic miR-21 Silencing Ameliorates Diabetic Kidney Disease in Mice. Mol. Ther. 25 (1), 165–180. 10.1016/j.ymthe.2016.08.001 28129112 PMC5363308

[B50] Kong A. P. S. Xiao K. Choi K. C. Wang G. Chan M. H. M. Ho C. S. (2012). Associations between microRNA (miR-21, 126, 155 and 221), Albuminuria and Heavy Metals in Hong Kong Chinese Adolescents. Clin. Chim. Acta 413 (13-14), 1053–1057. 10.1016/j.cca.2012.02.014 22405870

[B51] Kopp J. B. Anders H.-J. Susztak K. Podestà M. A. Remuzzi G. Hildebrandt F. (2020). Podocytopathies. Nat. Rev. Dis. Prim. 6 (1), 68. 10.1038/s41572-020-0196-7 32792490 PMC8162925

[B52] Kouri N. M. Stangou M. Lioulios G. Mitsoglou Z. Serino G. Chiurlia S. (2021). Serum Levels of miR-148b and Let-7b at Diagnosis May Have Important Impact in the Response to Treatment and Long-Term Outcome in IgA Nephropathy. Jcm 10 (9), 1987. 10.3390/jcm10091987 34063140 PMC8125269

[B53] Krol J. Loedige I. Filipowicz W. (2010). The Widespread Regulation of microRNA Biogenesis, Function and Decay. Nat. Rev. Genet. 11 (9), 597–610. 10.1038/nrg2843 20661255

[B54] Lai K. N. Leung J. C. K. Chan L. Y. Y. Saleem M. A. Mathieson P. W. Lai F. M. (2008). Activation of Podocytes by Mesangial-Derived TNF-α: Glomerulo-Podocytic Communication in IgA Nephropathy. Am. J. Physiology-Renal Physiology 294 (4), F945–F955. 10.1152/ajprenal.00423.2007 18256312

[B55] Lai K. N. Tang S. C. W. Schena F. P. Novak J. Tomino Y. Fogo A. B. (2016). IgA Nephropathy. Nat. Rev. Dis. Prim. 2, 16001. 10.1038/nrdp.2016.1 27189177

[B56] Lazzeri E. Romagnani P. (2015). Differentiation of Parietal Epithelial Cells into Podocytes. Nat. Rev. Nephrol. 11 (1), 7–8. 10.1038/nrneph.2014.218 25421831

[B57] Lee H. W. Khan S. Q. Khaliqdina S. Altintas M. M. Grahammer F. Zhao J. L. (2017). Absence of miR-146a in Podocytes Increases Risk of Diabetic Glomerulopathy via Up-Regulation of ErbB4 and Notch-1. J. Biol. Chem. 292 (2), 732–747. 10.1074/jbc.M116.753822 27913625 PMC5241746

[B58] Lee R. C. Feinbaum R. L. Ambros V. (1993). The *C. elegans* Heterochronic Gene Lin-4 Encodes Small RNAs with Antisense Complementarity to Lin-14. Cell. 75 (5), 843–854. 10.1016/0092-8674(93)90529-y 8252621

[B59] Leung A. K. Wong A. H. Barg S. S. (2017). Proteinuria in Children: Evaluation and Differential Diagnosis. Am. Fam. Physician 95 (4), 248–254. Available at: https://www.aafp.org/pubs/afp/issues/2017/0215/p248.html 28290633

[B60] Li C. Shi J. Zhao Y. (2018). MiR‐320 Promotes B Cell Proliferation and the Production of Aberrant Glycosylated IgA1 in IgA Nephropathy. J. Cell. Biochem. 119 (6), 4607–4614. 10.1002/jcb.26628 29266359

[B61] Li H. Chen Z. Chen W. Li J. Liu Y. Ma H. (2021a). MicroRNA-23b-3p Deletion Induces an IgA Nephropathy-like Disease Associated with Dysregulated Mucosal IgA Synthesis. J. Am. Soc. Nephrol. 32 (10), 2561–2578. 10.1681/asn.2021010133 34479967 PMC8722801

[B62] Li J. Liu B. Xue H. Zhou Q. Q. Peng L. (2017). miR-217 Is a Useful Diagnostic Biomarker and Regulates Human Podocyte Cells Apoptosis via Targeting TNFSF11 in Membranous Nephropathy. BioMed Res. Int. 2017, 1–9. 10.1155/2017/2168767 PMC5682891 29214160

[B63] Li Y. Xia M. Peng L. Liu H. Chen G. Wang C. (2021b). Downregulation of miR-214-3p A-ttenuates M-esangial H-ypercellularity by T-argeting PTEN-mediated JNK/c-Jun S-ignaling in IgA N-ephropathy. Int. J. Biol. Sci. 17 (13), 3343–3355. 10.7150/ijbs.61274 34512151 PMC8416718

[B64] Liang Y. Zhao G. Tang L. Zhang J. Li T. Liu Z. (2016). MiR-100-3p and miR-877-3p Regulate Overproduction of IL-8 and IL-1β in Mesangial Cells Activated by Secretory IgA from IgA Nephropathy Patients. Exp. Cell. Res. 347 (2), 312–321. 10.1016/j.yexcr.2016.08.011 27542871

[B65] Lin C.-L. Lee P.-H. Hsu Y.-C. Lei C.-C. Ko J.-Y. Chuang P.-C. (2014). MicroRNA-29a Promotion of Nephrin Acetylation Ameliorates Hyperglycemia-Induced Podocyte Dysfunction. J. Am. Soc. Nephrol. 25 (8), 1698–1709. 10.1681/asn.2013050527 24578127 PMC4116051

[B66] Liu D. Liu F. Wang X. Qiao Y. Pan S. Yang Y. (2018). MiR-130a-5p Prevents Angiotensin II-Induced Podocyte Apoptosis by Modulating M-type Phospholipase A2 Receptor. Cell. Cycle 17 (21-22), 2484–2495. 10.1080/15384101.2018.1542901 30394845 PMC6342077

[B67] Liu F. Chen J. Li Z. Meng X. (2022). Recent Advances in Epigenetics of Age-Related Kidney Diseases. Genes 13 (5), 796. 10.3390/genes13050796 35627181 PMC9142069

[B68] Liu Y. Li H. Liu J. Han P. Li X. Bai H. (2017). Variations in MicroRNA-25 Expression Influence the Severity of Diabetic Kidney Disease. J. Am. Soc. Nephrol. 28 (12), 3627–3638. 10.1681/asn.2015091017 28923913 PMC5698056

[B69] Long J. Wang Y. Wang W. Chang B. H. J. Danesh F. R. (2011). MicroRNA-29c Is a Signature microRNA under High Glucose Conditions that Targets Sprouty Homolog 1, and its *In Vivo* Knockdown Prevents Progression of Diabetic Nephropathy. J. Biol. Chem. 286 (13), 11837–11848. 10.1074/jbc.M110.194969 21310958 PMC3064234

[B70] Lu M. Wang C. Yuan Y. Zhu Y. Yin Z. Xia Z. (2015). Differentially Expressed microRNAs in Kidney Biopsies from Various Subtypes of Nephrotic Children. Exp. Mol. Pathology 99 (3), 590–595. 10.1016/j.yexmp.2015.10.003 26481277

[B71] Luyckx V. A. Perico N. Somaschini M. Manfellotto D. Valensise H. Cetin I. (2017). A Developmental Approach to the Prevention of Hypertension and Kidney Disease: a Report from the Low Birth Weight and Nephron Number Working Group. Lancet 390 (10092), 424–428. 10.1016/s0140-6736(17)30576-7 28284520 PMC5884413

[B72] Maas R. J. Deegens J. K. Smeets B. Moeller M. J. Wetzels J. F. (2016). Minimal Change Disease and Idiopathic FSGS: Manifestations of the Same Disease. Nat. Rev. Nephrol. 12 (12), 768–776. 10.1038/nrneph.2016.147 27748392

[B73] Mathieson P. W. (2011). The Podocyte as a Target for Therapies-New and Old. Nat. Rev. Nephrol. 8 (1), 52–56. 10.1038/nrneph.2011.171 22045242

[B74] Maurer B. Stanczyk J. Jüngel A. Akhmetshina A. Trenkmann M. Brock M. (2010). MicroRNA-29, a Key Regulator of Collagen Expression in Systemic Sclerosis. Arthritis & Rheumatism 62 (6), 1733–1743. 10.1002/art.27443 20201077

[B75] Medjeral-Thomas N. R. Lawrence C. Condon M. Sood B. Warwicker P. Brown H. (2020). Randomized, Controlled Trial of Tacrolimus and Prednisolone Monotherapy for Adults withDe NovoMinimal Change Disease. Clin. J. Am. Soc. Nephrol. 15 (2), 209–218. 10.2215/cjn.06180519 31953303 PMC7015084

[B76] Mellis D. Caporali A. (2018). MicroRNA-based Therapeutics in Cardiovascular Disease: Screening and Delivery to the Target. Biochem. Soc. Trans. 46 (1), 11–21. 10.1042/bst20170037 29196609

[B77] Ming L. Ning J. Ge Y. Zhang Y. Ruan Z. (2019). Excessive Apoptosis of Podocytes Caused by Dysregulation of microRNA‐182‐5p and CD2AP Confers to an Increased Risk of Diabetic Nephropathy. J Cell. Biochem. 120 (10), 16516–16523. 10.1002/jcb.28911 31131477

[B78] Mishra A. Ayasolla K. Kumar V. Lan X. Vashistha H. Aslam R. (2018). Modulation of Apolipoprotein L1-microRNA-193a axis Prevents Podocyte Dedifferentiation in High-Glucose Milieu. Am. J. Physiology-Renal Physiology 314 (5), F832–f843. 10.1152/ajprenal.00541.2017 PMC6031922 29357419

[B79] Mundel P. Reiser J. (2010). Proteinuria: an Enzymatic Disease of the Podocyte? Kidney Int. 77 (7), 571–580. 10.1038/ki.2009.424 19924101 PMC4109304

[B80] Mundel P. Shankland S. J. (2002). Podocyte Biology and Response to Injury. J. Am. Soc. Nephrol. 13 (12), 3005–3015. 10.1097/01.asn.0000039661.06947.fd 12444221

[B81] Munyentwali H. Bouachi K. Audard V. Remy P. Lang P. Mojaat R. (2013). Rituximab Is an Efficient and Safe Treatment in Adults with Steroid-dependent Minimal Change Disease. Kidney Int. 83 (3), 511–516. 10.1038/ki.2012.444 23325085

[B82] Nagata M. (2016). Podocyte Injury and its Consequences. Kidney Int. 89 (6), 1221–1230. 10.1016/j.kint.2016.01.012 27165817

[B83] Nasr S. H. Kopp J. B. (2020). COVID-19-Associated Collapsing Glomerulopathy: An Emerging Entity. Kidney Int. Rep. 5 (6), 759–761. 10.1016/j.ekir.2020.04.030 32368701 PMC7196556

[B84] Ollero M. Sahali D. (2015). Inhibition of the VEGF Signalling Pathway and Glomerular Disorders. Nephrol. Dial. Transpl. 30 (9), 1449–1455. 10.1093/ndt/gfu368 25480873

[B85] Pawluczyk I. Z. A. Didangelos A. Barbour S. J. Er L. Becker J. U. Martin R. (2021). Differential Expression of microRNA miR-150-5p in IgA Nephropathy as a Potential Mediator and Marker of Disease Progression. Kidney Int. 99 (5), 1127–1139. 10.1016/j.kint.2020.12.028 33417998

[B86] Peired A. Angelotti M. L. Ronconi E. la Marca G. Mazzinghi B. Sisti A. (2013). Proteinuria Impairs Podocyte Regeneration by Sequestering Retinoic Acid. J. Am. Soc. Nephrol. 24 (11), 1756–1768. 10.1681/asn.2012090950 23949798 PMC3810076

[B87] Puelles V. G. van der Wolde J. W. Wanner N. Scheppach M. W. Cullen-McEwen L. A. Bork T. (2019). mTOR-Mediated Podocyte Hypertrophy Regulates Glomerular Integrity in Mice and Humans. JCI Insight 4 (18), e99271. 10.1172/jci.insight.99271 PMC6795295 31534053

[B88] Qi H. Fu J. Luan J. Jiao C. Cui X. Cao X. (2020). miR-150 Inhibitor Ameliorates Adriamycin-Induced Focal Segmental Glomerulosclerosis. Biochem. Biophysical Res. Commun. 522 (3), 618–625. 10.1016/j.bbrc.2019.11.096 31787235

[B89] Qiu C. Huang S. Park J. Park Y. Ko Y.-A. Seasock M. J. (2018). Renal Compartment-specific Genetic Variation Analyses Identify New Pathways in Chronic Kidney Disease. Nat. Med. 24 (11), 1721–1731. 10.1038/s41591-018-0194-4 30275566 PMC6301011

[B90] Ramezani A. Devaney J. M. Cohen S. Wing M. R. Scott R. Knoblach S. (2015). Circulating and Urinary microRNA Profile in Focal Segmental Glomerulosclerosis: a Pilot Study. Eur. J. Clin. Invest. 45 (4), 394–404. 10.1111/eci.12420 25682967 PMC4903079

[B91] Reidy K. Kang H. M. Hostetter T. Susztak K. (2014). Molecular Mechanisms of Diabetic Kidney Disease. J. Clin. Invest. 124 (6), 2333–2340. 10.1172/jci72271 24892707 PMC4089448

[B92] Roberts I. S. D. (2014). Pathology of IgA Nephropathy. Nat. Rev. Nephrol. 10 (8), 445–454. 10.1038/nrneph.2014.92 24861083

[B93] Rodrigues J. C. Haas M. Reich H. N. (2017). IgA Nephropathy. Clin. J. Am. Soc. Nephrol. 12 (4), 677–686. 10.2215/cjn.07420716 28159829 PMC5383386

[B94] Ronco P. Beck L. Debiec H. Fervenza F. C. Hou F. F. Jha V. (2021). Membranous Nephropathy. Nat. Rev. Dis. Prim. 7 (1), 69. 10.1038/s41572-021-00303-z 34593809

[B95] Ronconi E. Sagrinati C. Angelotti M. L. Lazzeri E. Mazzinghi B. Ballerini L. (2009). Regeneration of Glomerular Podocytes by Human Renal Progenitors. J. Am. Soc. Nephrol. 20 (2), 322–332. 10.1681/asn.2008070709 19092120 PMC2637058

[B96] Rosenberg A. Z. Kopp J. B. (2017). Focal Segmental Glomerulosclerosis. Clin. J. Am. Soc. Nephrol. 12 (3), 502–517. 10.2215/cjn.05960616 28242845 PMC5338705

[B97] Ruggenenti P. Fervenza F. C. Remuzzi G. (2017). Treatment of Membranous Nephropathy: Time for a Paradigm Shift. Nat. Rev. Nephrol. 13 (9), 563–579. 10.1038/nrneph.2017.92 28669992

[B98] Schober A. Blay R. M. Saboor Maleki S. Zahedi F. Winklmaier A. E. Kakar M. Y. (2021). MicroRNA-21 Controls Circadian Regulation of Apoptosis in Atherosclerotic Lesions. Circulation 144 (13), 1059–1073. 10.1161/circulationaha.120.051614 34233454

[B99] Serino G. Pesce F. Sallustio F. De Palma G. Cox S. N. Curci C. (2016). In a Retrospective International Study, Circulating miR-148b and Let-7b Were Found to Be Serum Markers for Detecting Primary IgA Nephropathy. Kidney Int. 89 (3), 683–692. 10.1038/ki.2015.333 26581012

[B100] Serino G. Sallustio F. Cox S. N. Pesce F. Schena F. P. (2012). Abnormal miR-148b Expression Promotes Aberrant Glycosylation of IgA1 in IgA Nephropathy. J. Am. Soc. Nephrol. 23 (5), 814–824. 10.1681/asn.2011060567 22362909 PMC3338289

[B101] Sha W. G. Shen L. Zhou L. Xu D.-y. Lu G. Y. (2015). Down-regulation of miR-186 Contributes to Podocytes Apoptosis in Membranous Nephropathy. Biomed. Pharmacother. 75, 179–184. 10.1016/j.biopha.2015.07.021 26382839

[B102] Shankland S. J. (2006). The Podocyte's Response to Injury: Role in Proteinuria and Glomerulosclerosis. Kidney Int. 69 (12), 2131–2147. 10.1038/sj.ki.5000410 16688120

[B103] Shi S. Yu L. Chiu C. Sun Y. Chen J. Khitrov G. (2008). Podocyte-selective Deletion of Dicer Induces Proteinuria and Glomerulosclerosis. J. Am. Soc. Nephrol. 19 (11), 2159–2169. 10.1681/asn.2008030312 18776119 PMC2573016

[B104] Szeto C.-C. Li P. K.-T. (2014). MicroRNAs in IgA Nephropathy. Nat. Rev. Nephrol. 10 (5), 249–256. 10.1038/nrneph.2014.50 24709842

[B105] Tan K. Chen J. Li W. Chen Y. Sui W. Zhang Y. (2013). Genome-wide Analysis of microRNAs Expression Profiling in Patients with Primary IgA Nephropathy. Genome 56 (3), 161–169. 10.1139/gen-2012-0159 23659700

[B106] Tonneijck L. Muskiet M. H. A. Smits M. M. van Bommel E. J. Heerspink H. J. L. van Raalte D. H. (2017). Glomerular Hyperfiltration in Diabetes: Mechanisms, Clinical Significance, and Treatment. J. Am. Soc. Nephrol. 28 (4), 1023–1039. 10.1681/asn.2016060666 28143897 PMC5373460

[B107] Torban E. Braun F. Wanner N. Takano T. Goodyer P. R. Lennon R. (2019). From Podocyte Biology to Novel Cures for Glomerular Disease. Kidney Int. 96 (4), 850–861. 10.1016/j.kint.2019.05.015 31420194

[B108] Trajkovski M. Hausser J. Soutschek J. Bhat B. Akin A. Zavolan M. (2011). MicroRNAs 103 and 107 Regulate Insulin Sensitivity. Nature 474 (7353), 649–653. 10.1038/nature10112 21654750

[B109] Treiber T. Treiber N. Meister G. (2019). Regulation of microRNA Biogenesis and its Crosstalk with Other Cellular Pathways. Nat. Rev. Mol. Cell. Biol. 20 (1), 5–20. 10.1038/s41580-018-0059-1 30228348

[B110] Trionfini P. Benigni A. (2017). MicroRNAs as Master Regulators of Glomerular Function in Health and Disease. J. Am. Soc. Nephrol. 28 (6), 1686–1696. 10.1681/asn.2016101117 28232619 PMC5461805

[B111] Trionfini P. Benigni A. Remuzzi G. (2015). MicroRNAs in Kidney Physiology and Disease. Nat. Rev. Nephrol. 11 (1), 23–33. 10.1038/nrneph.2014.202 25385286

[B112] van Zandwijk N. Pavlakis N. Kao S. C. Linton A. Boyer M. J. Clarke S. (2017). Safety and Activity of microRNA-Loaded Minicells in Patients with Recurrent Malignant Pleural Mesothelioma: a First-In-Man, Phase 1, Open-Label, Dose-Escalation Study. Lancet Oncol. 18 (10), 1386–1396. 10.1016/s1470-2045(17)30621-6 28870611

[B113] Vivarelli M. Massella L. Ruggiero B. Emma F. (2017). Minimal Change Disease. Clin. J. Am. Soc. Nephrol. 12 (2), 332–345. 10.2215/cjn.05000516 27940460 PMC5293332

[B114] Waldman M. Crew R. J. Valeri A. Busch J. Stokes B. Markowitz G. (2007). Adult Minimal-Change Disease: Clinical Characteristics, Treatment, and Outcomes. Clin. J. Am. Soc. Nephrol. 2 (3), 445–453. 10.2215/cjn.03531006 17699450

[B115] Wang G. Kwan B. C.-H. Lai F. M.-M. Chow K.-M. Li P. K.-T. Szeto C.-C. (2011). Elevated Levels of miR-146a and miR-155 in Kidney Biopsy and Urine from Patients with IgA Nephropathy. Dis. Markers 30 (4), 171–179. 10.3233/dma-2011-076610.1155/2011/304852 21694443 PMC3825242

[B116] Wang L. Wang J. Wang Z. Zhou J. Zhang Y. (2021). Higher Urine Exosomal miR-193a Is Associated with a Higher Probability of Primary Focal Segmental Glomerulosclerosis and an Increased Risk of Poor Prognosis Among Children with Nephrotic Syndrome. Front. Cell. Dev. Biol. 9, 727370. 10.3389/fcell.2021.727370 34708038 PMC8542839

[B117] Wang X. Lin B. Nie L. Li P. (2017). microRNA-20b Contributes to High Glucose-Induced Podocyte Apoptosis by Targeting SIRT7. Mol. Med. Rep. 16 (4), 5667–5674. 10.3892/mmr.2017.7224 28849008

[B118] Webster A. C. Nagler E. V. Morton R. L. Masson P. (2017). Chronic Kidney Disease. Lancet 389 (10075), 1238–1252. 10.1016/s0140-6736(16)32064-5 27887750

[B119] Wei C. El Hindi S. Li J. Fornoni A. Goes N. Sageshima J. (2011). Circulating Urokinase Receptor as a Cause of Focal Segmental Glomerulosclerosis. Nat. Med. 17 (8), 952–960. 10.1038/nm.2411 21804539 PMC4089394

[B120] Wen L. Zhao Z. Xiao J. Wang Z. He X. Birn H. (2018). Renal miR-148b Is Associated with Megalin Down-Regulation in IgA Nephropathy. Biosci. Rep. 38 (6), BSR20181578. 10.1042/bsr20181578 30355654 PMC6239259

[B121] Wiggins R.-C. (2007). The Spectrum of Podocytopathies: a Unifying View of Glomerular Diseases. Kidney Int. 71 (12), 1205–1214. 10.1038/sj.ki.5002222 17410103

[B122] Williams A. M. Jensen D. M. Pan X. Liu P. Liu J. Huls S. (2022). Histologically Resolved Small RNA Maps in Primary Focal Segmental Glomerulosclerosis Indicate Progressive Changes within Glomerular and Tubulointerstitial Regions. Kidney Int. 101 (4), 766–778. 10.1016/j.kint.2021.12.030 35114200 PMC8940673

[B123] Wu J. Zheng C. Fan Y. Zeng C. Chen Z. Qin W. (2014). Downregulation of microRNA-30 Facilitates Podocyte Injury and Is Prevented by Glucocorticoids. J. Am. Soc. Nephrol. 25 (1), 92–104. 10.1681/asn.2012111101 24029422 PMC3871768

[B124] Wu J. Zheng C. Wang X. Yun S. Zhao Y. Liu L. (2015). MicroRNA-30 Family Members Regulate Calcium/calcineurin Signaling in Podocytes. J. Clin. Invest. 125 (11), 4091–4106. 10.1172/jci81061 26436650 PMC4639992

[B125] Wu L. Zhang X. Luo L. Li X. Liu Y. Qin X. (2021). Altered Expression of Serum miR‐106a, miR‐19b, miR‐17, and PTEN in Patients with Idiopathic Membranous Nephropathy. J. Clin. Lab. Anal. 35 (4), e23737. 10.1002/jcla.23737 33745222 PMC8059741

[B126] Wyatt R. J. Julian B. A. (2013). IgA Nephropathy. N. Engl. J. Med. 368 (25), 2402–2414. 10.1056/NEJMra1206793 23782179

[B127] Xiao B. Wang L.-N. Li W. Gong L. Yu T. Zuo Q.-F. (2018). Plasma microRNA Panel Is a Novel Biomarker for Focal Segmental Glomerulosclerosis and Associated with Podocyte Apoptosis. Cell. Death Dis. 9 (5), 533. 10.1038/s41419-018-0569-y 29748623 PMC5945632

[B128] Yang X. Wu D. Du H. Nie F. Pang X. Xu Y. (2017). MicroRNA-135a Is Involved in Podocyte Injury in a Transient Receptor Potential Channel 1-dependent Manner. Int. J. Mol. Med. 40 (5), 1511–1519. 10.3892/ijmm.2017.3152 28949388 PMC5627871

[B129] Yates L. A. Norbury C. J. Gilbert R. J. C. (2013). The Long and Short of microRNA. Cell. 153 (3), 516–519. 10.1016/j.cell.2013.04.003 23622238

[B130] Zha F. Bai L. Tang B. Li J. Wang Y. Zheng P. (2019). MicroRNA‐503 Contributes to Podocyte Injury via Targeting E2F3 in Diabetic Nephropathy. J Cell. Biochem. 120 (8), 12574–12581. 10.1002/jcb.28524 30834596

[B131] Zhai Y. Qi Y. Long X. Dou Y. Liu D. Cheng G. (2019). Elevated hsa‐miR‐590‐3p Expression Down‐regulates HMGB2 Expression and Contributes to the Severity of IgA Nephropathy. J. Cell. Mol. Med. 23 (11), 7299–7309. 10.1111/jcmm.14582 31557418 PMC6815813

[B132] Zhang C. Zhang W. Chen H.-M. Liu C. Wu J. Shi S. (2015). Plasma microRNA-186 and Proteinuria in Focal Segmental Glomerulosclerosis. Am. J. Kidney Dis. 65 (2), 223–232. 10.1053/j.ajkd.2014.07.013 25218681

[B133] Zhang K. Sun W. Zhang L. Xu X. Wang J. Hong Y. (2018). miR-499 Ameliorates Podocyte Injury by Targeting Calcineurin in Minimal Change Disease. Am. J. Nephrol. 47 (2), 94–102. 10.1159/000486967 29448244

[B134] Zhang W. Zhang C. Chen H. Li L. Tu Y. Liu C. (2014). Evaluation of microRNAs miR-196a, miR-30a-5P, and miR-490 as Biomarkers of Disease Activity Among Patients with FSGS. Clin. J. Am. Soc. Nephrol. 9 (9), 1545–1552. 10.2215/cjn.11561113 25107948 PMC4152819

[B135] Zhao B. Li H. Liu J. Han P. Zhang C. Bai H. (2016). MicroRNA-23b Targets Ras GTPase-Activating Protein SH3 Domain-Binding Protein 2 to Alleviate Fibrosis and Albuminuria in Diabetic Nephropathy. J. Am. Soc. Nephrol. 27 (9), 2597–2608. 10.1681/asn.2015030300 26839366 PMC5004638

[B136] Zhao J. Liu Z. (2020). Treatment of Nephrotic Syndrome: Going beyond Immunosuppressive Therapy. Pediatr. Nephrol. 35 (4), 569–579. 10.1007/s00467-019-04225-7 30904930

[B137] Zhdanova O. Srivastava S. Di L. Li Z. Tchelebi L. Dworkin S. (2011). The Inducible Deletion of Drosha and microRNAs in Mature Podocytes Results in a Collapsing Glomerulopathy. Kidney Int. 80 (7), 719–730. 10.1038/ki.2011.122 21544061 PMC3246347

[B138] Zheng Z. Hu H. Tong Y. Hu Z. Cao S. Shan C. (2018). MiR-27b Regulates Podocyte Survival through Targeting Adenosine Receptor 2B in Podocytes from Non-human Primate. Cell. Death Dis. 9 (11), 1133. 10.1038/s41419-018-1178-5 30429458 PMC6235956

[B139] Zhou G. Zhang X. Wang W. Zhang W. Wang H. Xin G. (2019). Both Peripheral Blood and Urinary miR-195-5p, miR-192-3p, miR-328-5p and Their Target Genes PPM1A, RAB1A and BRSK1 May Be Potential Biomarkers for Membranous Nephropathy. Med. Sci. Monit. 25, 1903–1916. 10.12659/msm.913057 30865617 PMC6427931

[B140] Zhou L. Liu Y. (2015). Wnt/β-catenin Signalling and Podocyte Dysfunction in Proteinuric Kidney Disease. Nat. Rev. Nephrol. 11 (9), 535–545. 10.1038/nrneph.2015.88 26055352 PMC4869701

[B141] Zhou W. Dong S. Chen Z. Li X. Jiang W. (2022). New Challenges for microRNAs in Acute Pancreatitis: Progress and Treatment. J. Transl. Med. 20 (1), 192. 10.1186/s12967-022-03338-2 35509084 PMC9066850

[B142] Zhou Z. Wan J. Hou X. Geng J. Li X. Bai X. (2017). MicroRNA-27a Promotes Podocyte Injury via PPARγ-Mediated β-catenin Activation in Diabetic Nephropathy. Cell. Death Dis. 8 (3), e2658. 10.1038/cddis.2017.74 28277542 PMC5386567

